# LncRNA 93358 Aggravates the Apoptosis of Myocardial Cells After Ischemia‐Reperfusion by Mediating the PI3K/AKT/mTOR Pathway

**DOI:** 10.1002/jbt.70085

**Published:** 2024-12-09

**Authors:** Jiumei Cai, Zhiwei Zhang, Lingling Chen, Xiaoping Wang, Yiming Zhong, Dongyang Xie, Wei Liao

**Affiliations:** ^1^ Department of Cardiology First Affiliated Hospital of Gannan Medical University Ganzhou China; ^2^ Department of Pediatric Cardiology Guangdong Cardiovascular Institute, Guangdong Provincial People's Hospital Guangzhou China

**Keywords:** H9c2 cells, ischemia‐reperfusion, LncRNA 93358, PI3K/AKT/mTOR pathway

## Abstract

To investigate the impact of LncRNA 93358 on ischemia‐reperfusion induced myocardial cell apoptosis and the underlying mechanism. After being subjected to hypoxia for 4 h, three models of hypoxia‐reoxygenation (H/R) with reoxygenation times of 8, 16, and 24 h were established. The expression of LncRNA 93358 was detected by qPCR, and the most suitable conditions were selected for subsequent experiments. The LncRNA 93358 knockout rat myocardial cells were established by transfecting with shRNA‐93358 and identified by the RT‐PCR assay, followed by constructing the in vitro H/R model. H/R myocardial cells were treated with blank medium (Model), shRNA‐NC (LncRNA 93358 NC), shRNA‐93358 (LncRNA 93358 knock), and shRNA‐93358 + LY294002 (LncRNA 93358 knockout+LY294002), respectively. Normal myocardial cells treated with blank medium was taken as the control group. The cell cycle and apoptosis were analyzed by the flow cytometry. The level of cellular SOD and MDA was measured by the ELISA assay. The expression level of LncRNA 93358 was determined by the RT‐PCR assay and Western blot assay was utilized to evaluate the expression level of AKT1, p‐AKT1, mTOR, p‐mTOR, bcl‐2, and Bax. Compared to control, the expression of LncRNA 93358 in H9C2 cells was significantly increased under hypoxic conditions for 4 h followed by reoxygenation for 8 h/16 h. Moreover, the expression of LncRNA 93358 was relatively higher under hypoxic conditions for 4 h followed by reoxygenation for 16 h. Compared to control, significantly lower p‐mTOR/mTOR and p‐AKT1/AKT1 level was observed in the model group, accompanied by the elevated MDA level, declined SOD level, increased apoptotic rate, enhanced arrest at S phase, upregulated Bax, and downregulated Bcl‐2. Compared to the model and LncRNA 93358 NC group, the expression level of p‐mTOR/mTOR and p‐AKT1/AKT1 was significantly promoted in the LncRNA 93358 knock group, accompanied by the declined MDA level, increased SOD level, reduced apoptotic rate, increased arrest at G0/G1 phase, downregulated Bax, and upregulated Bcl‐2, which were dramatically reversed in the LncRNA 93358 knockout+LY294002 group. LncRNA 93358 aggravated the apoptosis of myocardial cells after ischemia‐reperfusion by mediating the PI3K/AKT/mTOR pathway.

## Graphical abstract

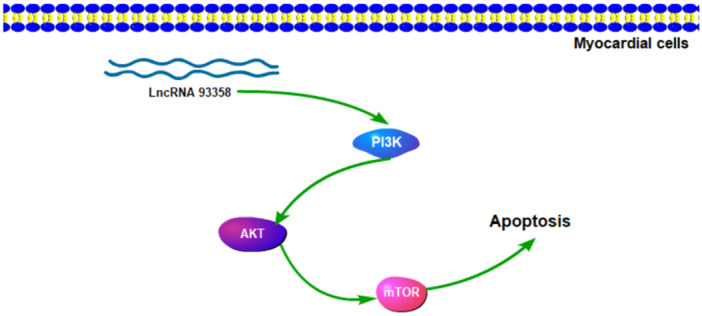

## 1 Introduction

In recent years, the morbidity of acute myocardial infarction (AMI) in China has been increasing annually, which triggers the attention from experts and scholars widely [1]. Strategies for the treatment of AMI in clinic mainly include surgery, thrombolytic therapy, interventional therapy, and so forth, which show a certain therapeutic effect. However, it is reported that the injuries on myocardial tissues will be aggravated under the treatment strategies against AMI, which is named as reperfusion injury [2, 3]. Reperfusion injury further aggravates the severity of myocardial injury in patients and poses a serious threat to the prognosis of patients. Therefore, alleviating myocardial cell ischemia‐reperfusion injury has become a hot topic in multiple clinical studies [4, 5]. PI3K/AKT signaling is a critical pathway, transduction proteins generated from which are involved in the regulation on the biological activity of cardiomyocytes [6]. The progression of apoptosis and cell cycle can be repressed by the activation of PI3K/Akt/mTOR signaling pathway, mainly manifested by phosphorylation of related targets. The phosphorylation level is closely associated with the degree of antiapoptosis [7].

Long noncoding RNAs (LncRNAs) are a category of noncoding RNAs that exceed 200 nucleotides, which are involved in regulating gene expressions from epigenetic, transcription, and posttranslational levels. LncRNAs are discovered to be differentially expressed and involved in the regulation in different types of diseases, including coronary heart disease (CHD), diabetes, and nervous system diseases, and so forth. For incidence, LncRNA ANRIL is found to be related to the processing of cardiovascular disease (CVD) [8]. Gong et al. [9] found that LncRNA MALAT1 promoted apoptosis of myocardial cells after myocardial infarction by targeting miR‐144‐3p and the apoptosis of myocardial cells was facilitated by the upregulation of LncRNA MALAT1.

In the preliminary study, LncRNA 93358, also known as LncRNA plasmacytoma variant translocation 1 (PVT1), was screened out by high‐throughput sequencing and identified to be highly expressed in AMI rats and the cell apoptosis in myocardium could be repressed by the knockdown of LncRNA 93358. It has been found that lncRNA PVT1 is upregulated in rat cardiomyocytes induced by H/R [10]. Whether the regulatory mechanism of LncRNA 93358 in controlling apoptosis of cardiomyocytes is mediated through the PI3K/AKT/mTOR signaling pathway remains unknow. To verify the hypothesis, in the present study, LY294002, an inhibitor of PI3K/AKT/mTOR signaling pathway, was used to explore the underlying mechanism for the regulatory effects of LncRNA 93358 on AMI.

## 2 Materials and Methods

### 2.1 Cells and Treatments

Rat myocardial cell line, H9c2 cells, were purchased from BeNa Culture Collection (BNCC) and cultured in DMEM medium containing 10% FBS and 1% Penicillin/Streptomycin under the condition of 37°C and 5% CO_2_.

### 2.2 The H/R Condition in H9c9 Cells Was Explored

H9C2 cells were cultured under normal oxygen conditions (containing 5% CO_2_ and 21% O_2_) at 37°C. In the hypoxia culture experiment, cells were placed in a hypoxia incubator, where the oxygen concentration was adjusted to 1% (containing 5% CO_2_ and 1% O_2_). Following the experimental groups of hypoxia for 4 h and reoxygenation for 8 h/16 h/24 h, qPCR detection was conducted.

### 2.3 The Knockout of LncRNA 93358 in H9c2 Cells

Firstly, the gene sequence of LncRNA 93358 was downloaded from the website of NCBI. Two target sequences were analyzed and linked to the vector of (MCS‐EF1a‐Cas9‐FLAG‐P2A‐puro) and the target sequences were shown as below: 1: F‐GTTACGTGTCCCCGCTTTAT and R‐ATTATCCGTGCATATCGCGC; 2: CTAAGTAGCATTCTCGTCAT and ATCTTAGAGTACAGCACTAC. The vector was transfected into HEK293T cells together with the transfection reagent, Lipofectamine 3000 (Invitrogen, California, USA), to obtain the virus particles. The blank vector without target sequences as taken as the negative control (LncRNA 93358 NC). The collected virus particles were transfected into myocardial cells together with HitransG A.

### 2.4 I/R Modeling on Myocardial Cells and Grouping

Five groups were divided: Control, Model, LncRNA 93358 NC, LncRNA 93358 knockout, and LncRNA 93358 knockout+LY294002. Approximately 72 h post virus transfection, cells in control group was cultured under the normal condition. Cells in the remaining four groups were replaced with DMEM medium without glucose and placed in the tri‐gas incubator for 4 h, followed by replacing with the complete DMEM medium and reoxygenation for 16 h. Cells in the LncRNA 93358 knockout+LY294002 group were treated with 100 nM LY294002 simultaneously.

### 2.5 Real Time PCR

1 mL of RNAiso Plus reagent was added to cells. After thorough mixing, the supernatant was discarded after centrifugation. Total RNA precipitation was purified using a 75% ethanol solution, followed by obtaining RNAs for reverse transcription. The specific steps were performed according to the instructions of the HiScript II Q RT SuperMix Kit (Vazyme, China). Real‐time PCR detection was carried out following the experimental instructions of the 7500 Real‐Time PCR System (ABI, USA). The reaction conditions were as follows: two‐step PCR amplification, pre‐denaturation at 95°C for 30 s, reaction at 95°C for 5 s, annealing at 60°C for 31 s, for 40 cycles, followed by calculating gene expressions utilizing the 2^−ΔΔCt^ method. Table 1 showed sequences of primers.

**Table 1:** Sequences of the primers.

Genes	Sequences (5′−3′)
Lnc 93358F	GTTGCCCCATCCTCATCTC
Lnc 93358R	TCACAAGTCGGCGGTTCT
β‐actin F	GCCATGTACGTAGCCATCCA
β‐actin R	GAACCGCTCATTGCCGATAG

### 2.6 Western Blot Analysis Assay

RIPA lysis buffer containing 1% proteinase inhibitor was added to cells, which were then lysed on ice for 30 min. Then, the lysate was centrifuged at 13,500 r/min for 10 min at 4°C. Protein quantification was performed using the BCA kit (CWBIO, China), and equal amounts of protein were loaded for electrophoresis. Following electrophoresis, the membrane was transferred at a constant current of 200 mA in the electrophoresis buffer for 2 h. The PVDF membrane was then blocked with 5% nonfat milk for 1 h. Overnight incubation was carried out with primary antibodies at the following concentrations: AKT1 (1:800, Abcam, UK), GSDMD (1:500, Affinity, USA), TLR4 (1:500, proteintech, USA), p‐AKT1 (1:1000, Affinity, USA), mTOR (1:1000, CST, USA), p‐mTOR (1:1000, CST, USA), bcl‐2 (1:1000, ABclonal, USA), Bax (1:5000, proteintech, USA), and GAPDH (1: 2000, ZSGB‐BIO, China) at 4°C. After washing, secondary antibodies (1:2000, ZSGB‐BIO, China) were loaded and incubated for 1 h. Chemiluminescent detection was used for visualization, and Image J software was employed for gray value analysis of the experimental bands.

### 2.7 ELISA Assay

Following centrifugation, supernatants were collected. SOD activities and MDA (Elabscience, China) were evaluated by ELISA using the commercial kits, with instructions followed.

### 2.8 Apoptosis Detection

After implanting in plates, cells were cultured 37°C for 2 days. Following resuspending in serum‐free medium, 5 μL of Annexin V‐FITC solutions and 10 μL of PI solutions (MULTI SCIENCES, China) were loaded to the cell suspension, followed by a 10‐min incubation in the absence of light. Subsequently, cell suspension placed in the flow tube for apoptosis analysis in the NovoCyte system (ACEA Biosciences, China).

### 2.9 Cell Cycle Detection

Cell suspension of each group was centrifuged and precipitates were added with 1 mL DNA staining reagent and 10 μL permeabilization solution. After stirring for 5–10 s, cells were well mixed and incubated without light for 30 min, followed by being tested on NovoCyte (ACEA Biosciences, China) for cell cycle analysis.

### 2.10 Statistical Analysis

Data obtained was analyzed using the GraphPad software and presented as mean ± SD. The Student's *t*‐test was employed to compare data between two groups, while data among more than three groups were analyzed using the one‐way ANOVA method. A significance level of *p* < 0.05 was used to determine statistical significance.

## 3 Results

### 3.1 The Expression of LncRNA 93358 in Rat Cardiomyocyte H9C2 Cells Under H/R Conditions

**Figure 1:**
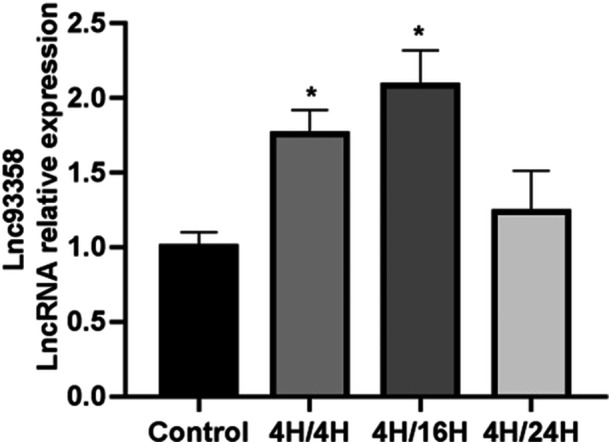
The expression levels of LncRNA 93358 knockdown in H9C2 cells under different H/R conditions were detected by qPCR. (*p < 0.05 vs. Control).

A H/R model of rat cardiomyocyte H9C2 cells was constructed, with hypoxia set for 4 h and reoxygenation for 8 h/16 h/24 h. The expression of LncRNA 93358 in H9C2 cells under H/R was detected using qPCR, with results shown in Figure 1. Compared to control, the expression of LncRNA 93358 in H9C2 cells was significantly increased under hypoxic conditions for 4 h followed by reoxygenation for 8 h/16 h. Moreover, the expression of LncRNA 93358 was relatively higher under hypoxic conditions for 4 h followed by reoxygenation for 16 h.

### 3.2 The Identification on the Knockout of LncRNA 93358

**Figure 2:**
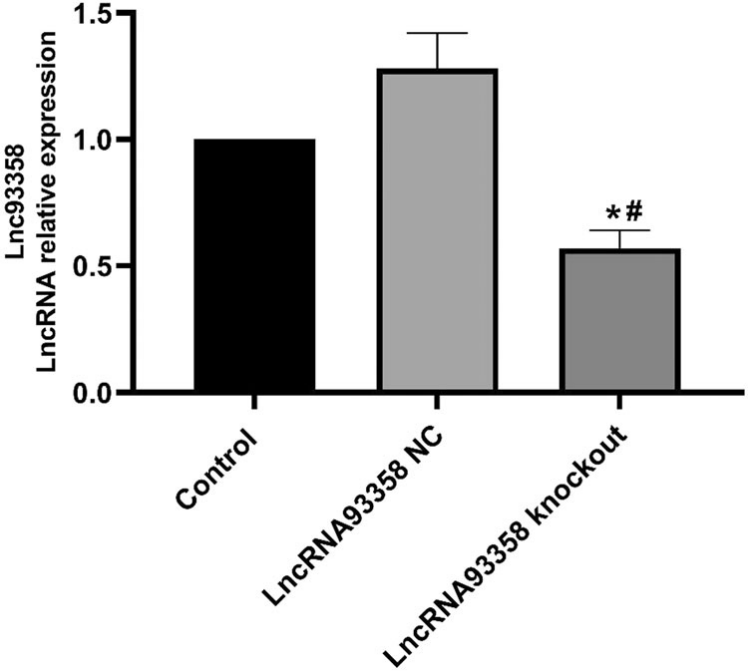
The expression level of LncRNA 93358 was evaluated by RT‐PCR (*p < 0.05 vs. Control, #*p* < 0.05 vs. LncRNA 93358 NC).

The expression level of LncRNA 93358 in virus transfected H9c2 cells was determined by the RT‐PCR assay. Compared to control and the LncRNA 93358 NC group, significantly lower expression level of LncRNA 93358 was observed in the LncRNA 93358 knockout group (Figure 2). These data indicated that the LncRNA 93358 knockout H9c2 cells were established successfully.

### 3.3 The Impact of LncRNA 93358 on the PI3K/AKT/mTOR Pathway

**Figure 3:**
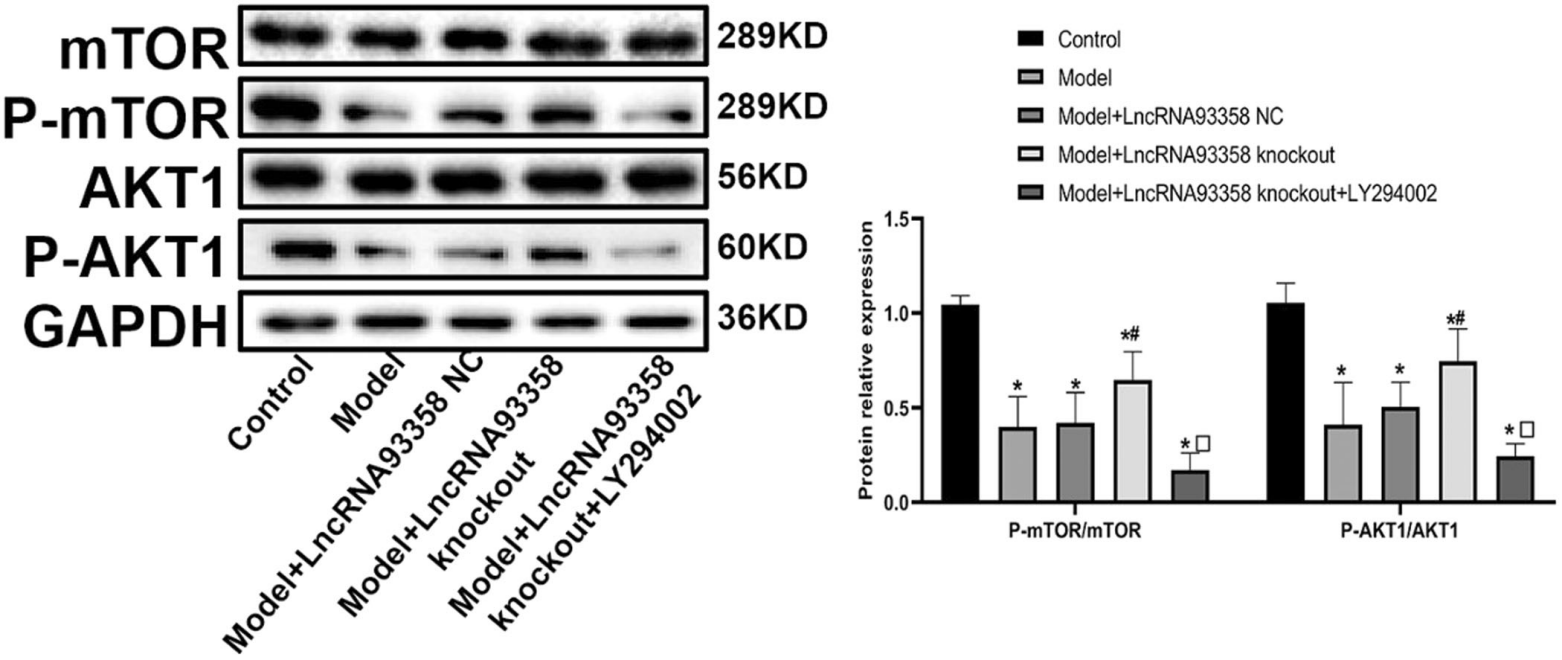
Western blot analysis was utilized to measure the expression level of key proteins in the PI3K/AKT/mTOR signal pathway in H9c2 cells. (*p < 0.05 vs. Control, #*p* < 0.05 vs. Model, Δ*p* < 0.05 vs. LncRNA 93358 NC, □*p* < 0.05 vs. LncRNA 93358 knockout).

Compared to control, p‐mTOR/mTOR and p‐AKT1/AKT1 levels were markedly repressed in the Model group, which were notably reversed in the LncRNA 93358 knockout group. Compared to the LncRNA 93358 knockout group, sharply lower p‐mTOR/mTOR and p‐AKT1/AKT1 levels were observed in the LncRNA 93358 knockout+LY294002 group (Figure 3).

### 3.4 The Impact of LncRNA 93358 on the State of Oxidative Stress in I/R Treated H9c2 Cells

**Figure 4:**
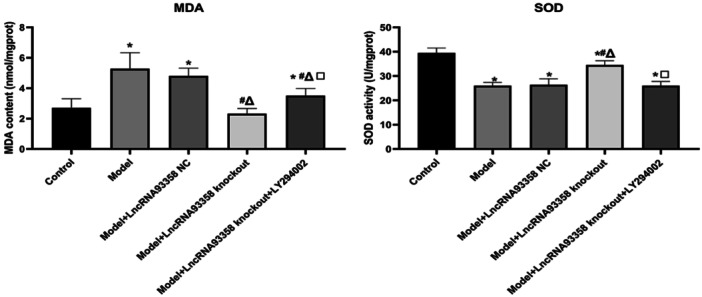
ELISA was used to measure the release of MDA and SOD in H9c2 cells. (*p < 0.05 vs. Control, #*p* < 0.05 vs. Model, Δ*p* < 0.05 vs. LncRNA 93358 NC, □*p* < 0.05 vs. LncRNA 93358 knockout).

To evaluate the effects of LncRNA 93358 on the oxidative stress in I/R treated H9c2 cells through regulating the PI3K/AKT/mTOR pathway, ELISA was utilized to detect the concentration of MDA and SOD in I/R treated H9c2 cells. As shown in Figure 4, compared to control, the release of MDA was significantly elevated and that of SOD was dramatically declined in the Model group, which were greatly reversed by the knockout of LncRNA 93358. In addition, compared to the LncRNA 93358 knockout group, the production of MDA was significantly promoted and that of SOD was greatly repressed in the LncRNA 93358 knockout+LY294002 group.

### 3.5 The Impact of LncRNA 93358 on the Cell Cycle of I/R Treated H9c2 Cells

**Figure 5:**
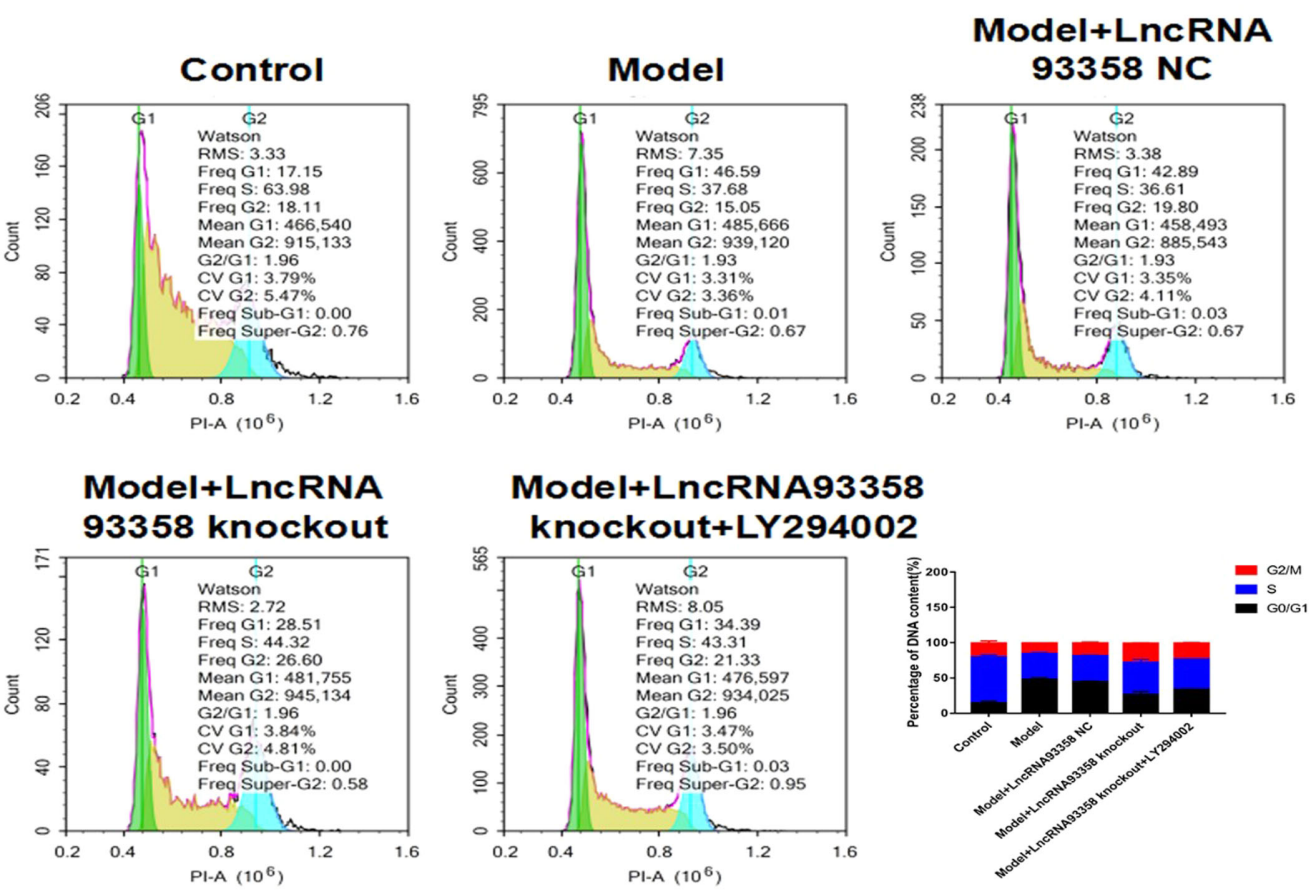
Flow cytometry was used to evaluate the cell cycle in H9c2 cells. (*p < 0.05 vs. Control, #*p* < 0.05 vs. Model, Δ*p* < 0.05 vs. LncRNA 93358 NC, □*p* < 0.05 vs. LncRNA 93358 knockout).

To explore the effect of LncRNA 93358 regulated PI3K/AKT/mTOR signaling pathway on the cell cycle of I/R treated H9c2 cells, flow cytometry was utilized to detect the apoptotic rate. As shown in Figure 5, compared to control, the DNA content in the Model group was arrested in the S phase. The DNA content was arrested in the G0/G1 phase. Furthermore, compared to the LncRNA93358 knockout group, the DNA content was arrested in the G2/M phase.

### 3.6 LncRNA 93358 Silencing Alleviated the Apoptosis in I/R Treated H9c2 Cells

**Figure 6:**
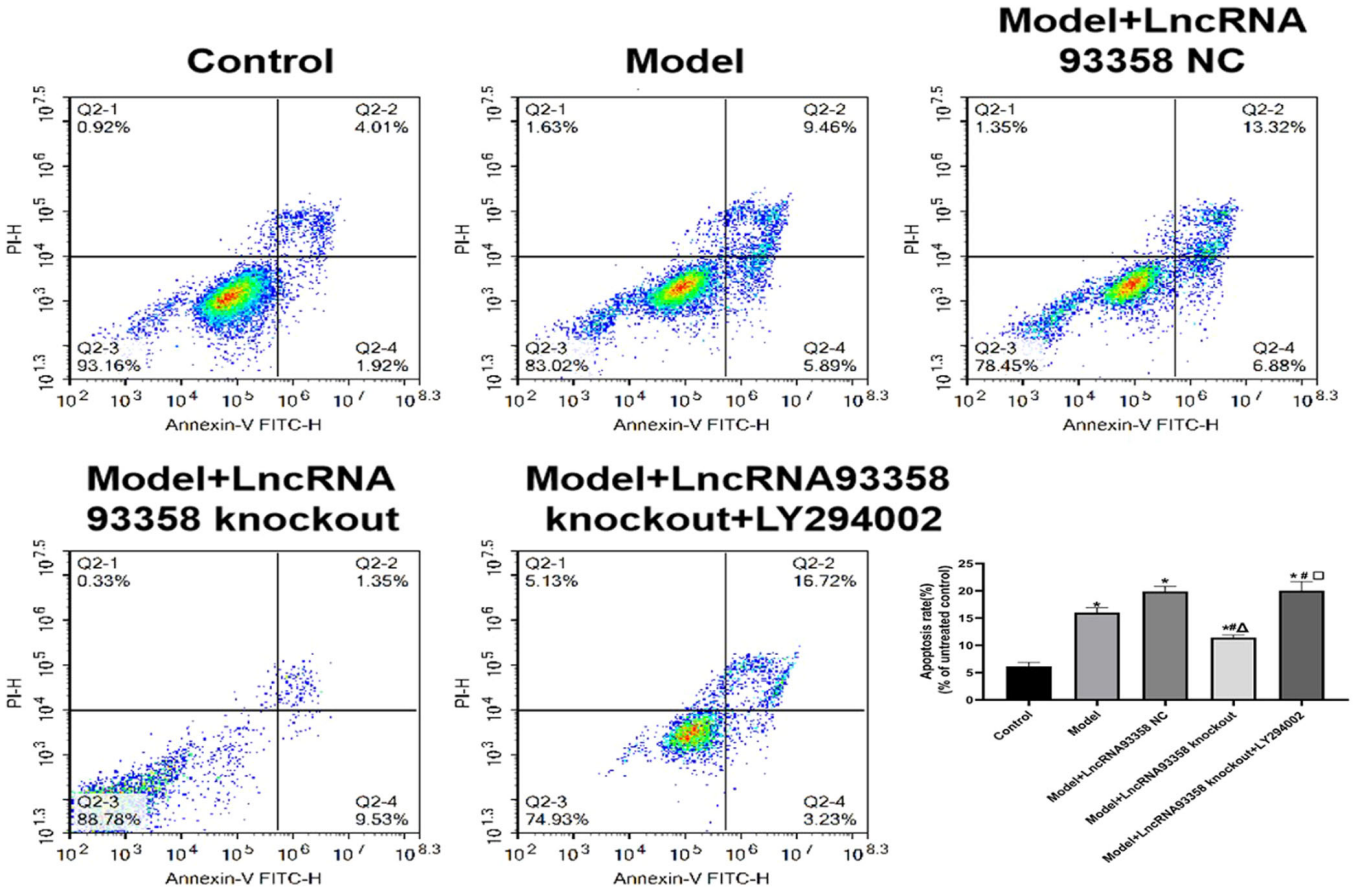
Flow cytometry was used to evaluate the apoptosis in H9c2 cells. (*p < 0.05 vs. Control, #*p* < 0.05 vs. Model, Δ*p* < 0.05 vs. LncRNA 93358 NC, □*p* < 0.05 vs. LncRNA 93358 knockout).

We further detected the apoptotic rate using the flow cytometry. As shown in Figure 6, compared to control, the apoptotic rate in the Model group was significantly elevated, which was greatly repressed in the LncRNA93358 knockout group. Compared to the LncRNA93358 knockout group, significantly higher apoptotic rate was observed in the LncRNA 93358 knockout+LY294002 group.

### 3.7 The Impact of LncRNA 93358 on the Expression Level of Apoptosis Proteins in I/R Treated H9c2 Cells

**Figure 7:**
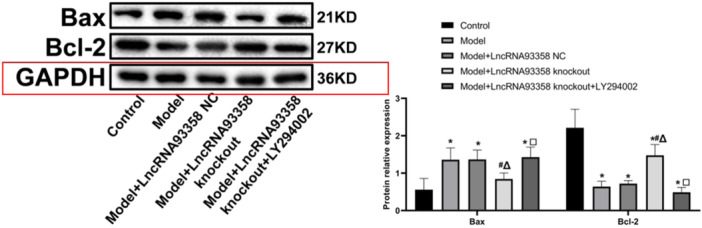
Western blot analysis was utilized to measure expression levels of Bax and Bcl‐2 in H9c2 cells. (*p < 0.05 vs. Control, #*p* < 0.05 vs. Model, Δ*p* < 0.05 vs. LncRNA 93358 NC, □*p* < 0.05 vs. LncRNA 93358 knockout).

Bax level was dramatically promoted and that of Bcl‐2 was sharply declined in the Model group, which were significantly reversed in the LncRNA93358 knockout group. Compared to the LncRNA93358 knockout group, significantly upregulated Bax and downregulated Bcl‐2 were observed in the LncRNA 93358 knockout+LY294002 group (Figure 7). These data further confirmed the antiapoptotic property of LncRNA 93358 in I/R treated H9c2 cells.

## 4 Discussion

Myocardial ischemia reperfusion injury (MIRI) is defined as the aggravated injury when the blood supply is restored after ischemia in myocardial tissues, which further triggers the damages on myocardial cells to elevate the mortality in myocardial infarction patients [11]. Seeking effective treatment strategies for MIRI and increasing the efficacy of reperfusion therapy against myocardial infarction are hotspots in current research.

Compared to gene‐protein coding, higher specificity is reported in the expression of LncRNAs in different tissues, implying that the role of lncRNA is intricately linked to the functional specificity of tissues [12]. In addition, similar to transcription factors, tissue‐specific expression patterns are observed in most LncRNAs. A recent study characterized LncRNAs highly expressed in myocardium by comparing RNA sequencing data from mouse myocardium, liver, and skin cells to identify 321 LncRNAs expressed in myocardium, among which 117 RNA transcripts showed abundant expression in the heart muscle. 67% of the LncRNAs among the 117 transcripts were also highly expressed in isolated heart muscle cells, suggesting that the majority of the highly expressed LncRNAs in mouse heart muscle mainly originated from cardiomyocytes [13]. Therefore, LncRNAs might be important regulators in the cardiac development and disease processes, along with new disease biomarkers or potential targets for cardiovascular disease and risk factors. Apart from overexpression of LncRNA 93358 (lncRNA PVT1) in H/R treated rat cardiomyocytes, overexpression of lncRNA PVT1 during the development of ovarian cancer can inhibit apoptosis [14]. LncRNA PVT1, located downstream of the oncogene MYC, has been proven to be closely related to the occurrence and progression of various tumors and acts as an oncogene. It has been found that lncRNA‐PVT1 can promote the proliferation and invasion of tumor cells [15]. There are also reports in the literature that lncRNA PVT1 exacerbates doxorubicin‐induced myocardial cell apoptosis by targeting the miR‐187‐3p/AGO1 axis [16]. In our preliminary study [17], the rat myocardial infarction model was constructed by coronary artery ligation and the differentially expressed gene LncRNA 93358 was screened by high‐throughput sequencing. Additionally, the apoptosis in myocardium was significantly induced by the downregulation of LncRNA 93358 in rats.

Studies have shown that the apoptosis will be repressed, the cell cycle will be facilitated, and the angiogenesis will be triggered by the activation of PI3K/Akt/m TOR signaling pathway [18]. In the processing of myocardial ischemia reperfusion, the phosphorylation of Akt will be triggered by the activation of PI3K to exert physiological functions [19]. Xi [20] found that the Akt phosphorylation was induced, the production of ROS was reduced, and the cell activity was enhanced by carvedilol through activating the PI3K/Akt/glycogen synthase kinase‐3β (GSK3β) pathway. It is reported that the activity of PI3K/Akt/mTOR signaling pathway can be regulated by LncRNAs. For incidence, the cell proliferation is inhibited and the apoptosis is induced by LncRNA GAS5 through regulating the PI3K/Akt/mTOR signaling pathway of triple negative breast cancer. In the present study, we found that the inactivated PI3K/AKT/mTOR signaling pathway in I/R treated H9c2 cells was significantly reversed by the downregulation of LncRNA 93358, which was verified by co‐introducing LY294002, an inhibitor of PI3K/AKT/mTOR pathway. These results suggested that the PI3K/AKT/mTOR signaling pathway in I/R myocardial cells could be mediated by LncRNA 93358.

MDA is mainly produced from ROS by attacking unsaturated fatty acids on biofilms and triggering lipid peroxidation. Moreover, oxidization on lipids, cell membrane proteins, and DNA will be induced by the excessive released ROS, which further contributes to a series of cell dysfunction or apoptosis and further aggravates tissue damage of ischemic testis. SOD is involved in cell growth and differentiation, which protects cells from oxidative damages by catalyzing superoxide free radical disproportionation reaction. The testicular damage can be effectively reduced by the antioxidant ability of SOD [21, 22]. PI3K/Akt/mTOR signaling pathway is involved in biological processes such as cell proliferation, differentiation and apoptosis, as well as the ROS‐mediated signal transduction. At the initial stage of oxidative stress, PI3K/Akt/mTOR signaling pathway will be activated to reduce the damage [23, 24, 25]. In the present study, we found that the oxidative stress was significantly induced in H9c2 cells by the treatment of I/R, which was greatly alleviated by the downregulation of LncRNA 93358. And the co‐introduction of LY294002 reversed the effects of the downregulation of LncRNA 93358, indicating that LncRNA 93358 might regulate the oxidative stress of I/R myocardial cells by mediating the PI3K/AKT/mTOR signaling pathway.

Studies have shown [26] that the angiogenesis and invasion of glioma cells can be repressed by silencing LncRNA HULC through inhibiting glioma cell proliferation, which induces apoptosis and blocks G1/S cell cycle through PI3K/Akt/mTOR signaling pathway. Thereby, tumor‐related genes involved in the above biological behaviors in human glioma U87MG and U251 cells were regulated. Bcl‐2 family is involved in the regulation of endogenous apoptosis pathways and it is reported that the expression of Bcl‐2 and Bax in the Bcl‐2 family is changed in myocardial cells with ischemia reperfusion, which further induces apoptosis [6]. These results showed that 3 days after myocardial infarction, the expression level of Bcl‐2 in AMI model group was significantly repressed, accompanied by the upregulation of Bax and increased apoptosis index of myocardial cells, indicating that Bcl‐2/BAX is an important pathway to induce apoptosis of myocardial cells. Under the state of myocardial infarction, apoptotic signals (ischemia, oxidative stress, etc.) will be generated in myocardial cells. The mitochondrial apoptosis pathway will be regulated by the proapoptotic gene Bax and antiapoptotic gene Bcl‐2 by regulating the permeability of mitochondrial outer membrane to release substances, such as cytochrome C and apoptosis‐inducing factor (AIF), from mitochondria into the cytoplasm, which further activate Caspase‐9 to participate in the initiation of apoptosis. As a consequence, Caspase‐3, the downstream executor of apoptosis, is lastly activated to impact the DNA replication and degrade apoptosis inhibitory proteins, which finally contributes to the apoptosis of cardiomyocytes [27, 28, 29, 30]. In the present study, the upregulated Bax and downregulated Bcl‐2 in the I/R treated H9c2 cells were significantly reversed by silencing LncRNA93358, accompanied by the decreased apoptotic rate and cell cycle arrested at G0/G1 phase. The effects of LncRNA93358 knockout on the apoptosis in I/R treated H9c2 cells were significantly reversed by LY294002, accompanied by cell cycle arrested at G2/M phase. These data indicated that LncRNA 93358 might regulate the apoptosis of I/R myocardial cells by mediating the PI3K/AKT/mTOR signaling pathway.

The study on the impact of LncRNA 93358 on myocardial cell apoptosis induced by I/R and its underlying mechanisms provides significant insights into the pathophysiology of AMI and potential therapeutic targets. The findings suggest that LncRNA 93358 plays a crucial role in mediating the PI3K/AKT/mTOR pathway, which is intricately linked to the regulation of apoptosis and cell survival in cardiomyocytes. The upregulation of LncRNA 93358 under hypoxic conditions followed by reoxygenation indicates its potential involvement in the adaptive response of myocardial cells to I/R injury. This is further supported by the observation that knocking down LncRNA 93358 leads to a significant decrease in apoptotic rate and an increase in the cell survival markers, such as the reduced expression of Bax and increased expression of Bcl‐2. These results are in line with previous studies that have shown the critical role of Bcl‐2 family proteins in the regulation of the intrinsic apoptosis pathway, where Bax promotes apoptosis and Bcl‐2 inhibits it [31]. Moreover, the study's findings on the modulation of the PI3K/AKT/mTOR pathway by LncRNA 93358 are particularly noteworthy. The activation of this pathway is known to promote cell survival and inhibit apoptosis by phosphorylating downstream targets such as mTOR, which in turn affects cell metabolism, growth, and proliferation [32]. The reversal of these effects by the PI3K/AKT/mTOR pathway inhibitor LY294002 suggests that LncRNA 93358 might exert its proapoptotic effects through this pathway. The implications of these findings are twofold. Firstly, they provide a molecular understanding of the processes that contribute to myocardial cell death following AMI, which is essential for developing targeted therapies to reduce the severity of reperfusion injury. Secondly, the identification of LncRNA 93358 as a potential therapeutic target opens up new avenues for the development of novel treatments aimed at modulating the PI3K/AKT/mTOR pathway to protect myocardial cells from I/R injury.

However, there are some limitations in the present study. Firstly, our study utilized the H9c2 rat myocardial cell line, which may not fully represent the complexity of human myocardial cells. This could limit the generalizability of the findings to other cell types or human myocardial cells. Secondary, our study used LY294002, a PI3K/AKT/mTOR pathway inhibitor, to explore the mechanism of LncRNA 93358. Relying on a single inhibitor may not fully account for the complexity of the pathway and could overlook other potential mechanisms. Lastly, the present study was conducted in vitro, and the findings may not directly translate to in vivo settings or clinical scenarios due to the differences in biological complexity. In our future work, the in vivo validation studies will be conducted to further confirm the role of LncRNA 93358 in MIRI.

Taken together, our data revealed that LncRNA 93358 aggravated the apoptosis of myocardial cells after ischemia‐reperfusion by mediating the PI3K/AKT/mTOR pathway.

## Author Contributions

Jiumei Cai designed the research and wrote the original draft. Wei Liao is responsible for the conception, design of the research, and the obtaining funding. Jiumei Cai, Lingling Chen, and Xiaoping Wang performed experiments. Yiming Zhong, and Dongyang Xie performed the analysis. All authors read and approved the final manuscript.

## Conflicts of Interest

The authors declare there is no conflicts of interest regarding the publication of this paper.

## Supporting information

Supporting information.

Supporting information.

Supporting information.

## Data Availability

All data are available from the corresponding author if requested by the journal or the readers.
