# Supplementary figures and images for: LncRNA 93358 Aggravates the Apoptosis of Myocardial Cells After Ischemia‐Reperfusion by Mediating the PI3K/AKT/mTOR Pathway
Source: J Biochem Mol Toxicol. 2024 Dec 9;38(12):e70085. doi: 10.1002/jbt.70085 (PMC11626694; doi:10.1002/jbt.70085)

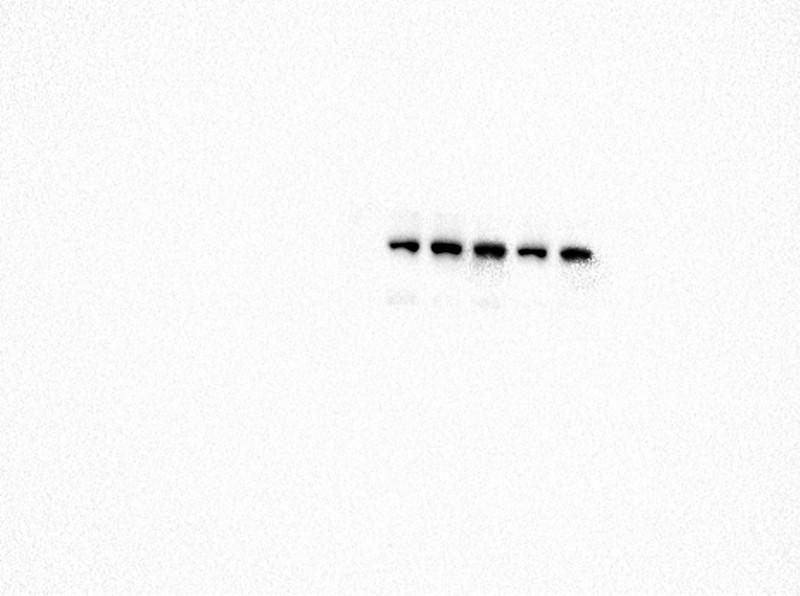

Supplement: Supplementary file 1 — Supporting information. [file JBT-38-e70085-s002.jpg]

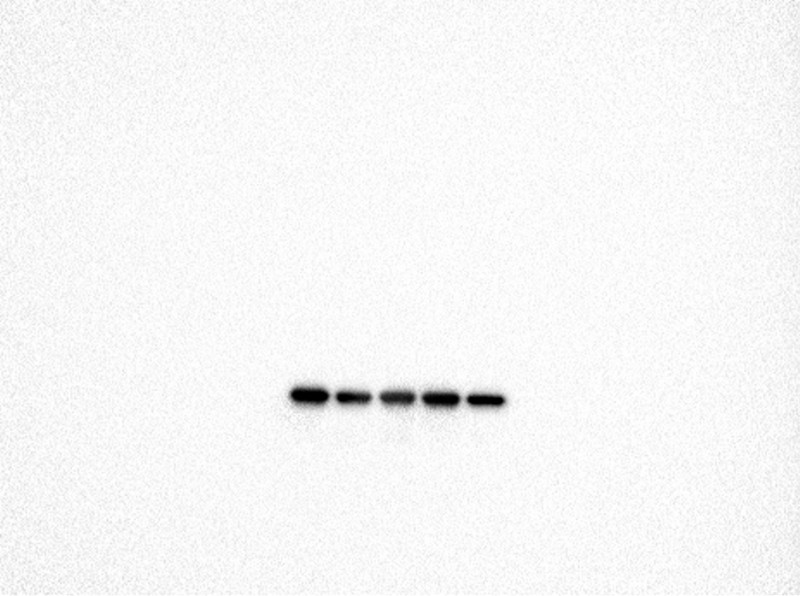

Supplement: Supplementary file 2 — Supporting information. [file JBT-38-e70085-s001.jpg]

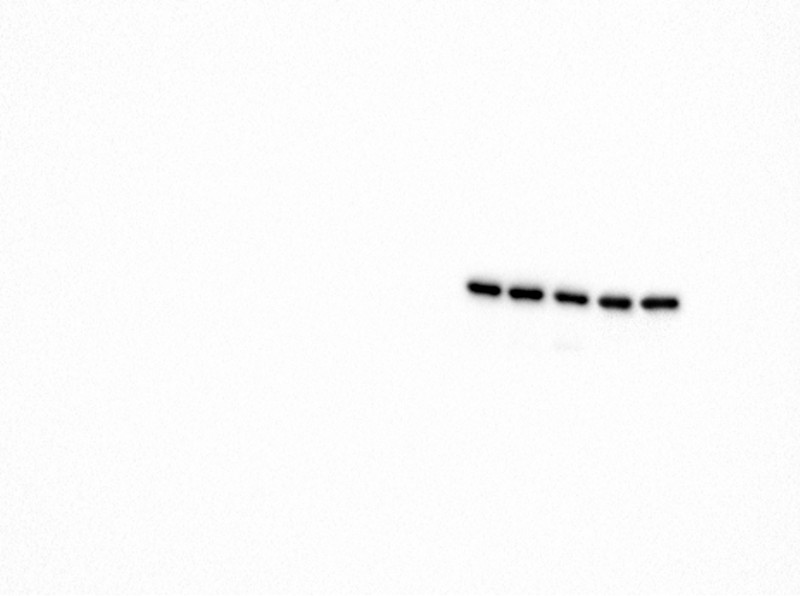

Supplement: Supplementary file 3 — Supporting information. [file JBT-38-e70085-s003.jpg]
